# “A mini home far away from home.” The Thai temple and women’s sense of safety in Finland

**DOI:** 10.3389/fpsyg.2024.1354068

**Published:** 2024-06-25

**Authors:** Mitra Härkönen

**Affiliations:** Faculty of Theology, Practical Theology, University of Helsinki, Helsinki, Finland

**Keywords:** Thai Buddhism, women, temple, migration, religious space, emotions, sense of safety

## Abstract

**Introduction:**

This article explores the significance of the Thai Buddhist temple for Thai women’s sense of safety in Finland. Despite the growing popularity of Buddhism in the country, research literature and media have largely ignored the religiosity of Thai women, focusing instead on negative stereotypes. The article highlights the importance of Buddhism and the temple in the lives of Thai women who have migrated to European countries and challenges biased portrayals of Thai women in previous research.

**Methods:**

Based on ethnographic research at the Thai temples in Finland and life story interviews with twelve Thai women.

**Results:**

The article argues that the temple plays a crucial role in promoting a sense of safety among Thai women by providing a sense of home, belonging, and meaning. The temple’s material and symbolic characteristics, in addition to its communality and relationality, work together to connect individuals with the temple’s religious significance, contributing to the women’s sense of security.

**Discussion:**

These findings speak for the fact that when facing the challenges of Thai women or other religious and ethical minorities, it is necessary to also look to their religion as a source of mental and spiritual well-being.

## 1 Introduction

There aren’t many newspaper articles that talk about the religious practices of Thai people. They just write about massage places, human trafficking, and berry-pickers; everything is about something negative. There is so much more here. Ordinary working people. And when it comes to sex workers, they are not bad people; they go to the temple and are very welcome to join. There are no restrictions that this temple is a holy place and that you cannot enter. The temple has no hierarchies, and no one’s background is looked at. It is a safe place. *Kaew, a Thai woman living in Finland*

In this article, I analyze the significance of the Thai Buddhist temple in Finland for Thai women’s sense of safety. Buddhism has become increasingly popular in Finland in recent years. People practicing Buddhism have risen from just over 10,000 in 2010 to around 30,000 in 2024. Additionally, there has been a noteworthy increase in the number of Buddhist organizations operating in the country, with around 30 new communities established in the 21st century alone (Härkönen, 2023a, 128–129). While Buddhism has become more and more popular among people of Finnish background, most of Finland’s Buddhist population are of Asian descent, and in recent years, they have come primarily from Thailand (Pauha and Martikainen, 2022; Härkönen, 2023b). Today, Thai Buddhism, along with Vietnamese Buddhism, is the largest and most vibrant form of Buddhism in Finland, with three established temples and thousands of practitioners. This is mainly due to Thai women living in the country (Härkönen, 2023b).

Even though Thai women are the primary supporters and promoters of Thai Buddhism in Finland, both research literature and popular media have mostly kept silent about the religiosity of Thai women living in European countries. Instead, the dominant discourse on them has been problem-oriented and has concentrated on the stereotypes of Thai women as sex workers or women as submissive victims of Western husbands or human trafficking (see Sirkkilä, 2005, 2015; Hartikainen, 2006; Lumio, 2011; Kuosmanen, 2012; Orava, 2014).

In this article, I aim to present a different perspective on Thai women immigrating to the Global North and settling down from the viewpoint of the Buddhist religion, especially of the Thai temple, *wat*. The temple and temple community play significant roles in Thai Buddhist lifestyles, centered around the home and temple as life’s most important points of reference (Sasiwongsaroj et al., 2012). Like in Thailand, Thai temples in Finland play a vital role in facilitating religious and communal gatherings and meritorious activities for Thai residents in the country. Furthermore, as a transnational space, the temple serves as a hub where Buddhism and Thainess can be upheld, maintained, and renewed, even far from home. Consequently, the temple acts as a center for numerous religious and social activities, symbolizing the perpetuity of Thai Buddhism and the Thai culture, identity, language, and people in a new homeland.

Based on ethnographic research conducted at Thai temples in Finland, as well as life story interviews with women of Thai origin, the article explores the significance of the Thai temple in promoting a sense of safety among Thai women who reside in Finland. Safety can be seen as having two dimensions: objective and subjective. The objective dimension of safety can be measured, while the subjective dimension is an individual’s feeling of security in their environment and its relationships (see Nilsen et al., 2004). In the article, I focus on the latter dimension of safety.

Research on the sense of safety can be found in various fields, such as psychology, sociology, criminology, urban planning, and architecture. As an ethnographer with an education in anthropology and the study of religions, my approach to the question at hand is data-driven and inductive. I have moved from the concrete and specific to the abstract and general, developing a theoretical understanding based on the data gathered (see Graneheim et al., 2017, 30). Thus, the goal has not been to test the suitability of the theory on the sense of safety based on the research material but rather to engage the theory in dialogue with the data in order to understand the multidimensionality of the phenomenon under study.

Drawing from the research data, I argue that a sense of safety at the temple is created through three main factors: material and symbolic means, communality and relationality, and the ultimate meaning the temple embodies. The temple’s material and symbolic aspects, such as its architecture, decorations, clothing, food, sounds, and smells, provide a *sense of being at home*. The social interactions at the temple among people who share the same cultural background and immigration experience strengthen this feeling and promote a *sense of belonging*. The temple’s material and non-material aspects remind women of the ultimate meaning of Buddhism, which is to overcome ignorance and suffering and achieve liberation. Thus, visiting the temple creates a sense of familiarity, comfort, and the feeling of being seen, heard, and accepted by others. This, along with feelings of relief, peace, and happiness promoted by the temple, can lead to a sense of security and emotional experience that the external environment and others are meeting one’s safety needs. Thus, to understand how the Thai temple can promote a sense of safety for Thai women in Finland, we must examine the temple as a transnational religious space, which is simultaneously physical, material, symbolic, and social.

I will start the article by discussing the limitations of previous research that has portrayed Thai women in a biased manner and will highlight the significance of Buddhism and the *wat* in the lives of numerous Thai women who have migrated. After that, I will present my theoretical framework, research materials, and methods. Finally, I will analyze the data using the materiality, relationality, and ultimate meaning approach in order to highlight how the temple serves as a source of security for many Thai women and how this sense of safety is fostered within the temple environment. In conclusion, I will discuss the key findings of the research.

## 2 From one-sided images to Thai women’s religious agency

### 2.1 Feminized Thai migration to Europe

Thai migration to Europe was minimal before the 1970s as Thailand had no colonial ties and there were no refugee-related flows to the Global North. However, (intimate) relations between Thais and Westerners were already established during the Vietnam War in the 1960s and 1970s, as Thailand was a major destination for the “rest and recreation” holidays for American soldiers. As a result, this generated the growth of the prostitution and entertainment business in Thailand (Esara, 2009; Suksomboon, 2012; Fresnoza-Flot, 2017). Currently, Thailand has become one of the most popular travel destinations in the world and in the Nordic countries, Thailand is one of the most popular travel destinations. According to the statistics, around 150,000 Finns visit Thailand every year (Finland in Thailand, n.d.; Statista, n.d.).

Today, Thai women are among the most popular marriage partners in transnational marriages with Western men (e.g., Fernbrant et al., 2017; Fresnoza-Flot, 2017; Fresnoza-Flot and Merla, 2018). In recent years, the spouse of Finnish men who have married a foreign citizen has most often been of either a Thai or Russian background. Due to the prevalence of marriages between Finnish men and Thai women, the majority (nearly 83%) of the more than 12,000 Thais living in Finland are women (Statistics Finland, 2022a,b,c). The situation is similar in all Nordic countries, as well as in many other European countries (Fernbrant et al., 2014, 2017; Fresnoza-Flot, 2017; Straiton et al., 2019).

Studies conducted on Thai women living in European countries have typically focused on their marriage motives, as well as how well they have adapted to their new environment and their overall state of well-being. According to research, Thai women find Western men ideal husbands because they are perceived as financially secure and trustworthy. It has been claimed that in Thai culture, the husband expresses his love through acknowledging and fulfilling his wife’s material needs and that the intimate relationship between the husband and the wife is of subordinate importance to this (Sirkkilä, 2005; Esara, 2009; Pravattiyagul, 2021). Thus, marriage between a Thai woman and a Western man is often understood in the framework of *hypergamy* in which a woman marries a person of a superior caste or class because this offers her and her family the opportunity to climb up the social ladder (e.g., Pravattiyagul, 2021). Some studies have, however, questioned the assumption that marriages are based either on money or on romantic love (Lapanun, 2012; Suksomboon, 2012). It is stressed that while marriages between Thai women and Western men take place in a context of various global asymmetries (such as wealth, power, resources, and status), they cannot be reduced to economic motives and hope for social mobility. Motives and reasons for (marriage) migration are not uniform but depend on various differences, such as class, age, and origin within Thailand (Suksomboon, 2012).

Despite the aforementioned differences, public discourse about Thai women in Europe is often generalizing and negative. Thai women are seen as exotic, submissive, sexually available, sexual slaves, or sex workers (Sirkkilä, 2005; Orava, 2014; Heikkilä and Rauhut, 2015; Fernbrant et al., 2017). Studies focusing on the integration and psychological well-being of Thai women in a new homeland have shown how harmful the stereotypes and prejudices are to them. The research has also shown that in addition to the prevailing prejudices, the lack of autonomy, discrimination, social isolation due to poor language skills, the lack of contact with others, and intimate partner violence can make Thai women especially vulnerable (Hartikainen, 2006; Lumio, 2011; Kuosmanen, 2012; Ihonen, 2013; Fernbrant et al., 2014, 2017; Pongthippat et al., 2018; Straiton et al., 2019). It is often believed (as suggested by Fernbrant et al., 2014) that the situation of Thai women improves over time. However, studies like the one conducted by Straiton et al. (2019) have shown that the continuous stress of balancing competing demands of work and transnational family life can lead to a decline in women’s health and well-being.

### 2.2 The unspoken religiosity of Thai women

Even though there is a growing number of studies on Thai women in transnational marriages, the research is silent about the role and significance of Thai Buddhism in women’s everyday (ethical) choices and practices. Since Buddhism does not exercise strict control over marriage to a partner of a different religion (e.g., Esara, 2009; Suksomboon, 2012), this may have given rise to the belief that Buddhism is irrelevant in other life choices as well. Furthermore, Buddhism in the West is often understood as a philosophical and psychological worldview and not as a religion with ethics, myths, and rituals. This idea of Buddhism echoes the widespread modernist interpretation of Buddhism (see McMahan, 2008). Finally, secularized Western society is often unable to see the far-reaching importance of religion in people’s lives.

Contrary to popular belief, it is important to note that religion holds significant importance: around 93–95 percent of Thais living in Thailand are Buddhists, and Buddhism is an integral part of the identity and everyday life of most Thais (e.g., Kitiarsa, 2010; Scott, 2017). This seems to be even more so for Thai women, who have been found to be more active lay practitioners of religion, the main supporters of the Thai Buddhist monastic institution, and the primary socializers of children into Buddhist ethics and everyday practices (e.g., Van Esterik, 1982; Lindberg Falk, 2007; Tsomo, 2020).

Research has shown that migration can change the meaning and the role of religion in many ways. Some give up their religion, while many others become more aware of their religious identity in a new environment. Moreover, religion is not only an expression of a religious identity but also an ethnic one, linked to the culture of the country of origin (Martikainen, 2004, 2013; McLoughlin, 2009; Martikainen and Sakaranaho, 2018). While some Thai women may give up Buddhism when they move to Finland, this seems uncommon. In fact, in their roles as lay practitioners and supporters of religion, Thai women have been key players in Finland as well. Women are the main importers, rooters, and supporters of Thai Buddhism here, and the *wat* and its permanent monks can measure the prosperity of Thai Buddhism (Härkönen, 2023b).

### 2.3 Thai Buddhist community in Finland

The first Thai Buddhist association was the Finnish-Thai Buddhist Association founded in 1994 by a group of active Thai women residing in Finland. These women used to meet regularly at a Thai restaurant in Helsinki, where they bonded over food, shared news, talked about their homesickness, and supported each other while taking care of their children. These meetings were crucial to the women, and they came up with the idea of creating a space where Thai people could come together to share their lives, joys, and sorrows. This proposed space would enable the women to maintain their country of origin’s culture while also allowing Finns to learn more about their culture. Given that Buddhism is an essential part of Thai culture and identity, they decided to establish a Buddhist temple (Härkönen, 2023b).

In 2000, a monk became Finland’s first Thai monk following a request by women and contributions from Thai monasteries in Sweden and Norway. To provide a residence and meeting place for the monks, a house was rented in Helsinki until 2002. Afterward, the community was able to purchase a property in Sipoo in the Helsinki capital region using the funds they had collected. Pra Mahanual became the abbot of the newly established Buddharam temple, which, however, soon became too small to accommodate the growing number of Thai attendees. In 2009, a new location in Nurmijärvi was discovered around 1 h drive from Helsinki. In 2017, the construction of the long wooden log temple building with an interior surface area of 400 m^2^ began. The new Wat Buddharama temple was completed in 2019 and officially opened in July 2022, after facing delays caused by the COVID-19 pandemic. The three-day celebration was attended by over a hundred monks and thousands of Thais from various parts of the world (Härkönen, 2023b).

Today, the Finnish-Thai Buddhist Association has approximately 1,200 members. In 2016, the community also registered a religious community called the Finnish Thai Buddhist Community, with over 600 members. However, a clearly larger number of people participate in the community’s activities, especially in the various Buddhist festivals organized by the community. The temple also serves, for example, Finns, Cambodians, Laotians, and Vietnamese (Härkönen, 2023b).

In 2006, Wat Phutta Tham Finland, a Thai Buddhist temple active in the Turku area of southwest Finland, was established. In the same year, a monk was invited to Finland from the Wat Dalarnavanaram temple in Dalarna, Sweden. Due to the lack of a permanent residence permit, he initially frequently traveled between Sweden and Finland. In 2011, he settled permanently in Finland after the association purchased a detached house, which was converted into the community temple known as Wat Phutta Tham that same year. There are around 350 members in the community, but more people visit the temple and attend the celebrations organized by the temple yearly (Härkönen, 2023b).

In 2018, the Wat Asokaram Finland Vipassana Meditation Center Association became the third Thai Buddhist association to be registered. Today, the community works under the name of Wat Pah Buddha Metta Thammatharo Finland. Its temple was established in Lohja, which is just an hour’s drive away from Helsinki. The temple follows the Thai forest monk tradition and is led by a forest monk from Thailand, similar to the temple in Turku (Härkönen, 2023b).

Thai Buddhism has a special place in Finland for several reasons. It is the country’s biggest and most active Buddhist school, and it is also a growing phenomenon. This is largely due to the marriage migration of Thai women, who make up the majority of Thai people living in Finland. As a result, Thai Buddhism is clearly dominated by women. Although the monks play a crucial role in Thai Buddhism, it would not be possible to sustain the temples without the support of women who contribute through donations, volunteering, and other forms of assistance. Despite this, Thai Buddhism and its temples remain relatively invisible in Finland, and this is, I argue because the religiosity of Thai women often goes unnoticed.

## 3 Constructing a sense of safety in religious space

### 3.1 Sense of safety

The concept of safety can have various meanings. It can be defined as freedom from danger and risks, or the condition of being safe from undergoing or causing hurt, injury, or loss. At its root, safety revolves around wholeness and health. The World Health Organization, for instance, defines safety as having two dimensions, an objective dimension, which can be seen as behavioral and environmental factors measured against external criteria, and a subjective dimension, which can be variously defined as the individual’s internal feelings or perceptions of being safe (Nilsen et al., 2004).

This article deals with the latter dimension of safety or a sense of safety. A sense of safety refers to an individual’s feeling of security and how they emotionally experience the external environment to meet their safety needs (Ratnayake, 2017; Collins and Guidry, 2018; Zou and Fang, 2020). Like any emotion, a person’s sense of safety is highly subjective, depending on various intrinsic factors such as personality traits, culture, nationality, risk tolerance, and socio-demographic background. However, an individual’s sense of safety is not solely based on subjective, intrinsic feelings, but it also depends heavily on the external environment and their interactions with others in this environment (Zou and Yu, 2022). Thus, in addition to the psychological and subjective dimensions, the sense of safety has social and objective dimensions (Nilsen et al., 2004; Ratnayake, 2017).

To comprehend how the Thai temple can create a feeling of safety for Thai women residing in Finland, it is important to explore its physical environment, symbolic value, and social relationships and how these influence an individual’s personal and subjective sense of security. In other words, we must examine the temple as a transnational religious space simultaneously physical, material, symbolic, and social.

### 3.2 Temple as a religious space

The temple and temple community have traditionally played significant roles in Thai Buddhist lifestyles, centered around the home and temple, which are life’s most important points of reference. The temple serves as a sacred space where the Buddha delivered his teachings and sermons to both monastics and laypeople. Buddhist temples have always been an important part of a community’s foundation. Whenever a village or community was founded, a temple would be constructed as a spiritual sanctuary for its members. Besides religious practices, Buddhist temples have provided secular activities since ancient times. Monks rely on alms offered by the laypeople, which promotes a close relationship between them. This close relationship, combined with religious obligations, has made social responsibility an essential obligation for monks. Today, Thai people in Thailand visit one of more than 37,000 temples in the country on Buddhist holidays and important festivals to make merit and wishes for themselves and their loved ones. People also go to the temple for family traditions such as birthdays, marriage ceremonies, and funerals (Sasiwongsaroj et al., 2012, 5–7).

While the temple is a religious space intended for religious and social purposes, it is not only that. The relationship between religion and space is more complex and multifaceted. Religious traditions have specific beliefs and practices related to the use and significance of physical space, but at the same time, space itself can also have an impact on religious beliefs and practices, as different environments can shape the way people experience and understand sacred or divine. Thus, material elements such as buildings, clothing, and other religious objects are not just passive containers of ready-made meaning, but they play an active role in producing religious meanings (Meyer, 2013 in Burchardt and Westendorp, 2018, 164). Religious objects and symbols impact our lived experiences. They are perceived and experienced by the human body in various ways, such as perception, feeling, memory, imagination, and practice. Thus, religion is not just interpreted and understood but also felt through touch, sound, smell, and sight (Hazard, 2013, 62–63; Morgan, 2017). The interplay between religious ideas and physical actions expressed through materiality produces religious experience and knowledge. As humans engage with “the divine,” supernatural powers, or sacred they shape religious spaces (Kilde, 2022, 3).

### 3.3 Religion and emotions

What is important here is that as a material, social, and religious space, the temple produces, conveys, and shapes emotions. Overall, religion and emotions are closely intertwined. Religious traditions cultivate emotions like love, compassion, and gratitude and religion can shape how individuals experience and interpret emotions such as guilt, shame, and fear. Religions promise to ease and improve one’s emotional state, and while not always, several researchers have shown that through religion, people can gain valuable social support and coping resources, which can contribute to their well-being. Religions can, for example, offer various ways to distance oneself from everyday emotions, and on the other hand, within a religious context, individuals may experience and convey emotions that remain suppressed in their daily lives. Religious settings can help individuals redefine social emotions while supporting societal emotional standards (Riis and Woodhead, 2010, 82, 115; Wu et al., 2021, 291).

Moreover, it has been proposed that religion’s strong influence on individuals’ health and well-being may be due to its ability to provide meaning. Religion is a perspective that people use to comprehend and interpret the world. As a result, it is closely related to the idea of meaning. Like other systems of meaning, religion impacts a person’s beliefs, goals, and emotions. However, religion is unique in its emphasis on the *sacred* and how this influences an individual’s beliefs, goals, and emotions (Park, 2005, 295, my emphasis).

While emotions are personal, they are also social and cultural. Thus, an individual’s relationships with others, cultural symbols, and the physical environment around her also shape them (Riis and Woodhead, 2010, 5, 52–53). The Thai temple, for instance, is not only a place for religious practices but also serves as a platform for social interactions and relationships. While human relationships and communality provided by the temple are crucial at home in Thailand, they hold a special value when experienced far away from home. As a transnational space, the temple allows people and monastic orders to connect across national borders. It is a place where individuals can come together to make new friends, share their life experiences, and provide support to one another through times of joy and sorrow.

In addition to social, emotions develop in relation to both material and symbolic aspects. Religion is a good example of this, as it usually involves numerous sacred objects and symbolic expressions with emotional significance. Expressions in material and immaterial forms, such as religious architecture, pictures, statues, music, coordinated bodily actions, and rituals, can help the participants experience different feelings and relationships. Religious objects may also act as emotional mediators between people, facilitating the expression of feelings that would otherwise be left uncommunicated (Riis and Woodhead, 2010, 96–97).

A sense of safety is an emotion that is developed through our relation to the environment and people around us. As with any emotion, a sense of safety is simultaneously biological, personal, and private, as well as relational, social, and cultural. Religion can play an important role in providing a sense of safety for individuals and communities. Many religious traditions offer a framework for understanding the nature of danger and protection and provide rituals, prayers, and other practices aimed at invoking “divine” protection and guidance. Additionally, religious communities often provide a sense of belonging and social support that can help individuals feel safer and more secure. Yet, it is important to note that religion is not always a source of safety, and in some cases, religious beliefs and practices can contribute to feelings of fear, guilt, or shame.

When examining the relationship between a religious space and a feeling of safety, it becomes apparent that a religious space, as a physical location, a religious and sacred space, and a social gathering place, can elicit different emotions, one of which may be a feeling of security. After introducing my materials and methods, I will examine how the temple can provide a sense of safety by incorporating and enhancing emotions through its symbolic and material features, communality and relationality, and religious meaning.

## 4 Materials and methods

### 4.1 Fieldwork and interviews

The article is based on ethnographic fieldwork at Thai Buddhist temples located in Finland and interviews with Thai women living in Finland. Between 2019 and 2023, I conducted fieldwork at the above-mentioned temples. I paid visits to Wat Phutta Tham Finland and the temples in Wat Asokaram Finland Vipassana Meditation Center and regularly spent time at the largest one, Wat Buddharama temple. During the fieldwork, I attended several Buddhist calendar festivals throughout this period, bringing hundreds of Thai women and sometimes their families to these temple venues. In addition, I visited the temples on ordinary days to observe temple life on both weekdays and weekends. The level of my participation varied from observing from a distance to taking part in everything I could. I, for example, participated in temple celebrations as a guest but also volunteered for small chores with other women. I spent dozens of hours at the temples during the four-year period.

In addition to participant observation at the temples, I interviewed 13 Thai women aged between 33 to 65 years who had been residing in Finland for seven to 32 years. There is approximately 18 h of interview material. I recruited most of the research participants from the temples I visited or used the snowball sampling technique. While many participants were actively involved in one or more temples, not all were. The interviews were conducted either in Finnish, English, or both. Most of the interviews were done at the women’s homes, where they kindly invited me. Two of the interviews I did on Zoom as requested by the research participants. Many interviews ended up being both intense and intimate.

The interviews were in-depth, and the women candidly shared their life stories, from their childhood and family life up until their present moment. Additionally, they discussed the role Buddhism and the temple played in their lives. Many of these women had come to Finland through marriage, while a few had come to study or work and only later found their Finnish spouses. During the time of the interview, six of the women were either married or in a long-term relationship with a Finnish man, while the rest were either divorced or widowed. The women came from different parts of Thailand, but most of them had some background in agriculture, and most described their childhood family living conditions as poor. Despite this, eight of the women had a degree in higher education either from Thailand or Finland. Nevertheless, none of them worked in their original profession received from Thailand. Instead, many had continued their studies in Finland or worked in fields that did not require higher education. The primary reason for not being able to use the education and work experience gained in Thailand was the language barrier, which was sometimes real but not always. Thus, even when fluent in English, many of the women could not find a job in Finland because their Finnish skills were inadequate.

### 4.2 Analysis

In ethnography, analysis begins in the field and ends with writing (Hammersley and Atkinson, 2007, 158). The long-term fieldwork made it possible for me to notice what was missing from the data and supplement the material. To analyze the data, I used a thematic approach, which involves identifying the key topics or themes that emerge from the data. Themes are recurring topics that appear in various forms throughout the data. The thematic analysis begins by reading the data, looking for patterns, and categorizing them. The next step is to identify all the data that relate to these patterns and combine them into sub-themes for a more detailed analysis. Once the data is organized by categories, it becomes possible to analyze the aspects that are most relevant to the research, such as the theme of safety in my own study. By systematically sifting through and comparing the data, it becomes possible to see the mutual relationships and internal structures of the categories. Finally, the categories that emerge from the analysis are often used to provide a description and/or explanation of the case or cases being investigated (Braun and Clarke, 2006; Hammersley and Atkinson, 2007, 162–166).

### 4.3 Ethics

The women spoke about their lives very openly, and some of the interviews lasted for hours. While I felt very privileged to hear the women’s stories, I am, at the same time, aware that many ethical questions follow from the women’s openness and the ethnographic method. For this reason, it has been essential to respect the dignity and integrity of the research subjects in all situations, including the interviews and ethnographic inquiry. It has thus been my top priority to treat the research subjects with the utmost respect. It has been important to ensure that no harm, whether physical, mental, social, or economic, is caused to them during the data gathering, storage, or when findings are reported in research publications (The Finnish Advisory Board on Research Integrity, 2019).

The temple is a public place where everyone is welcome to spend time. As a researcher, I was introduced to the people visiting the temple as a scholar interested in the religious practices of Thai Buddhist women. Nevertheless, when observing at the temples, I have taken care to write about the events and people in a way that individuals cannot be identified. Participation in the interviews was entirely voluntary and based on informed consent. I gave the participants information about the topic and the aims of the research, its duration, research methods, and how the research material is used and stored. Participants had the right to withdraw from the study at any time without any consequences. To protect the women interviewed, I use pseudonyms and do not specify their age, where they are from in Thailand, where they live in Finland, or how long exactly they have stayed here.

### 4.4 Epistemology and reliability

Methodological and ethical choices are closely linked to epistemological questions concerning the nature and limitations of knowledge (Moore and Sanders, 2006, 1). The reality that ethnographers document is a construction of accounts produced by the people they study. Data is thus shaped and constructed by the researcher, the subjects, and the research situation. Therefore, the purpose of qualitative research is not to gather pure data free from potential bias. Instead, the ethnographer should be aware of how the data is produced and constructed (Hammersley and Atkinson, 2007; see also Davidson Bremborg, 2011, 311). Qualitative interviews and life stories are also more constructionist than positivist and personal narratives should not be evaluated for reliability or validity (Warren, 2001, 83–84). Despite this, life stories can provide researchers with valuable information about social reality and a person’s place in the social order (Atkinson, 1998).

The ethnographer plays a crucial role in her research, and to ensure the accuracy of the research material, she needs to practice reflexivity and understand her position in relation to the research. In this study, there are significant differences between me and the research subjects, including ethnicity/nationality and sometimes educational and social background. I grew up in a secularized and individual-oriented Finland with a Lutheran upbringing. Additionally, I have no immigrant or Buddhist background. Hence, I acknowledge that despite my empathetic attitude towards my research subjects, I cannot fully understand and experience the situation of the women I am studying. I also recognize that there is always a power dynamic between the researcher and the research subjects, which I am mindful of throughout this research (see Harding and Norberg, 2005). The research process, from selecting a topic to collecting and analyzing data, considering the theoretical perspective, and interpreting results, has thus been extensively filtered through me. Furthermore, it is important to note that research findings cannot be easily generalized and automatically applied, for example to health and mental care. However, they provide valuable insights into the role of religion in the mental and psychological well-being of immigrants.

## 5 Results

### 5.1 Material environments and cultural symbols

The Wat Buddharama temple is located in the heart of the Finnish countryside and can be reached by car in about 1 h from Helsinki. There used to be a Finnish farm where the temple is now located. What is left of the former are a storehouse and an old yellow two-story wooden detached house on the plot, which serves as a residence for the monks and a venue for smaller gatherings. Walking towards the temple, one is surrounded by Finland’s national trees, the birches, which give the place a very Finnish appearance. Otherwise, the temple area is surrounded by forests and fields. The landscape changes with the seasons; the lush greenery of summer transforms into the vibrant colors of autumn and the snow-covered scenery of winter.

Despite the Finnish elements, the temple area has a strong connection with Thailand. At the entrance of the temple’s large parking area, there is a spirit house, and the pond in the temple courtyard is adorned with Buddha statues in various positions. A new, massive wooden log temple building blends in perfectly with the Finnish landscape, but it still has unique characteristics that distinguish it from typical Finnish architecture. Dozens of shoes are left on the temple’s large porch, where wind chimes tinkle in the breeze, inviting guests to enter.

The main hall’s interior is truly impressive. Although the wooden walls and vast windows with golden curtains overlooking the field landscape still bear a Finnish touch, the temple’s architecture and decoration leave no doubt that one has entered a Buddhist *wat*. The altar is the most striking feature, with the statue of Shakyamuni Buddha at its center. The arrangements of smaller statues, candles, flowers, fruits, and incense surround the principal Buddha statue. On the left side of the altar, there is a long podium for the monks to sit during the ceremonies. The ceiling’s border features a series of images depicting the phases of Buddha’s life from birth to *parinirvana*, or the nirvana after death. The front of the altar structure is lined with large carpets for laypeople to sit on, with chairs brought out during parties. On the right side, there are sofa groups where visitors can sit. At the other end of the temple complex, one can find a kitchen, storage rooms, social facilities, and an open office space. Between the more mundane and the altar areas, there are tables where the monks’ lunch is laid out daily on one round table, while other tables serve laypeople’s meals. Most of the temple’s items, from altar decorations and statues to furniture and textiles, were imported from Thailand.

The temple is open for visitation at any time for those who wish to pray, meditate, or have a conversation with the monks and other Thais. On a typical weekday, the atmosphere in the temple is both sacred and worldly. In the morning, a few laypeople come to the temple to bring or prepare food for the monks, who have their last meal of the day by noon. The remaining food is shared among the laypeople in attendance. Others clean, fix, and arrange the space inside and outside the temple. There is always something to do in the temple, and maintaining the temple would not be possible without these volunteers. The monks themselves also take care of some of the arrangements while occasionally stopping to chat with the laity. The vibe is relaxed and friendly, with people chatting and laughing. After lunch, most of the laypeople usually depart. Some are retired and head home, while others rush off to their evening shift at work.

Leading up to and during the celebration, the temple comes to life. The temple organizes annual Buddhist and cultural celebrations according to the lunar calendar. These include Magha Puja or “Sangha Day,” Visakha Puja or “Vesak Day” and Asalha Puja or “Dhamma Day,” Songkran or Thai New Year, Mother’s Day, and Loy Krathong Day or “Ancestors Day.” “Kathina Ceremony” celebrates the end of the three-month rains residence retreat and is the day when people offer the monks a set of robes. In addition, Father’s Day and New Year’s Eve are often celebrated.

Usually, these festivals and celebrations gather hundreds or, sometimes, even thousands of people of Thai background and their families in the temples. The temple’s parking lot is full of cars, and during big celebrations, orderlies are needed there to direct traffic. The party area extends from the temple complex to the yard, where food and drinks are often laid out. Stalls selling Thai food, clothes, and handicrafts dominate the courtyard area and the way to the temple. Especially in good weather, the atmosphere is like a market. People are socializing with each other, shopping, eating, and taking selfies. The yard is filled with activity as the monk’s teachings echo from the outdoor speakers.

As the monk’s speech takes place, some guests choose to listen outside while many others gather indoors. They are sitting on chairs or the floor, completely absorbed in the speech. Devotion is expressed through various gestures such as prostrations, raising the hands with palms pressed together, and sitting with legs tucked behind. The most basic act of devotion is paying homage in front of an image, typically of the Buddha, and offering flowers, incense, and candles. The ceremonies vary depending on the occasion and can include offering food, money, and robes, reciting texts appropriate for the event, and so on, all of which are carried out with appropriate rituals. One of the monks, or often one of the women, recites the formulas through a microphone, and the assembly follows. Although most people are familiar with the basic chants, some may have different levels of knowledge regarding chanting. However, the “Salutation to the Three Jewels” is well-known by everyone and is repeated three times. The celebration concludes with a shared lunch around noon, which is enjoyed after the monks have eaten. After lunch, the celebration can continue with activities such as Thai dancing, a beauty competition, or a lottery.

In a shared public ritual, all the elements work together to support each other. These include the sound world, such as monks’ recitation or joint recitation, the form of the ritual, the tranquility of religious imagery and temple architecture, the fragrance of incense, and the taste and smell of the food. These sensuous expressions and elements collaborate to elevate everyday life and foster a sense of religious community. The atmosphere is devoted and sacred, yet lively. People move around in the best Thai costumes, chatting with acquaintances in different Thai dialects and taking group selfies in front of the altar, which they can share on Facebook with their friends and relatives in Finland and Thailand. Volunteers collect donations and register them while others prepare food in the kitchen and set the tables. The aroma of food mixes with the fragrance of incense and perfumes. The religious architecture and images of the temple, as well as its prayer books, fabrics in the tablecloths, women’s dresses, and monks’ robes, all embody Thai religion and culture. The temple’s foods, smells, sounds, and language further contribute to its unique cultural identity. The temple is a space where Thainess can be fully experienced, celebrated, and renewed.

As a religious space, the temple is the material result of the ideas and activities of Thai people who settled in Finland. When the early Thai women formed the first Finnish-Thai Buddhist association, their aim was to establish a physical space for monks to reside and the Thais to practice Thai religious customs. Additionally, they hoped that the temple would serve as a place for Finns to learn about Thai culture and religion. Today, the temples in Finland are established physical places which display material objects and symbols of Thai Buddhism and culture and where religious practices are acted out. As such, the temple and its material aspects are important in carrying religious meanings that are reserved and negotiated in the new environment (Hazard, 2013; Kilde, 2022).

While the temple serves as a religious space to convey the symbolic meaning of Thai Buddhism, it is crucial to note that it also plays an active role in creating these meanings. As was argued above, religious items like buildings, clothing, and others not only contain religious meaning but also build religious meanings themselves. Humans experience religious symbols and objects through various senses and shape religious spaces as they interact with sacred or supernatural powers. Furthermore, the temple’s material and symbolic features create, convey, and facilitate emotions. Symbolic expressions, like religious architecture, pictures, statues, music, actions, and rituals, help people experience different feelings. Rituals, drama, and music objectify religious emotions, while other religious objects act as emotional mediators, facilitating uncommunicated feelings. Material objects, religious imagery, and symbolic elements shape personal experiences intertwined with autobiographies.

It is thus not surprising that many of the Thai women interviewed referred to the temple as “home”. For example, Chailai, who found a temple only after living in Finland for some years, said:

Had I known that there were other Thais here and a temple in Finland, I would have gone. Sometimes you want something familiar around you. This is like a mini home far away from home.

Boribun also mentioned home when she was talking about the temple:

In the temple, I feel comfort, I feel like I’m back at home.

The temple is a familiar space that evokes feelings of comfort, well-being, peace, and happiness. Like home, it is a place where women feel they can return when they encounter obstacles. Samorn, who had lived in Finland for nearly thirty years, said:

It’s a tradition, an old habit [to visit the temple]. It is good for the heart to go and pray there. To give food to the monks. It feels good. If bad things happen in life, it feels good to go there and pray.

Chatmanee, who has also lived in Finland for decades agreed with this when she said:

When you come to the temple, you feel good. Somewhere in the bottom of your heart, it makes you happy. I do not know how to tell you; it just feels so good inside. I cannot say if it’s the same for everyone, but for me, it just feels so good.

For these women, the temple reminded them of home, understood both as a physical or geographical location and as a space of belonging and identity (see Brickell and Datta, 2016). All of them were raised as Buddhists, and as a child, their families regularly took them to the temple, where they were taught Buddhist stories, values, and lifestyles. It was precisely this familiarity of physical material objects and cultural symbols that created a sense of home (see Wiles, 2008). While the women visiting the temple themselves embodied and conveyed Buddhist values and lifestyle, they also sensed the temple’s materiality and immateriality, which evoked an emotional memory of familiarity and homeliness. For many, this emotional experience of home was happy and comforting, although many found it difficult to put their emotions or experiences about the temple into words. When this sense of home is combined with the feeling of happiness, it is not far-fetched to claim that the temple, as a spatial and material, plays a significant role in the experience of security for many of these women.

### 5.2 Communality and relationality

Nevertheless, the temple provided the women with a sense of home, comfort, and safety not solely because of its physical features, material culture, and symbols but also because of the interactions and relationships that took place within it. In addition to its religious purpose, the temple also served an essential social role, as it has done throughout its history. Kaew, who had lived in Finland for more than twenty years, said:

When I moved to Finland, it was natural for me to look for a Thai temple. It feels like home, even though we are not relatives.

The feeling of home for Kaew at the temple was produced especially by relationships with other people of Thai background who felt as close as relatives. She continued to share her experience of losing a family member far away from home and the importance of other Thai people for this experience:

For example, yesterday, there was a woman who had lost her mother recently, and I immediately went to chat with her and got peer support. There’s no need to explain so much; we understand each other so well and how we feel. The fact that we are far away from Thailand and the feeling of guilt is enormous, not being able to care and help, and the fact that we are here [at the temple] and can at least do something brings the feeling of safety and peace. We hugged each other. We did not even ask each other’s names, but even though we immediately got peer support. I believe she left the temple satisfied, and so did I. You cannot get it anywhere else. Even though there is a crisis center here, the church, and all that in Finland. Losing a loved one is a crisis, everyone understands that. But it’s different. If you have your own temple, somehow, the monk’s support and words are quite strangely comforting. He just says, “Keep trying; this is life; everyone gets old and dies.” We already know this; it’s a fact. But when a monk says this, and you stop, think, and try to understand it, it somehow calms you down.

Kaew also described how the monk’s presence at her deceased relative’s funeral brought her a feeling of safety. She commented, “It felt like he was my relative”.

Some Thai people search for the temple soon after arriving in Finland, while others have lived in the country for some time before discovering it. Before finding the temple, many said that they missed the sense of community and culture that came with being in Thailand. Kamlai stated:

Before I went to the temple here for the first time, I had not socialized with Thai people. I missed Thainess. [At the temple,] we can speak our own language. It feels like being at home when we are together. We can eat our own traditional food and speak our own dialect. We return to our roots, to the place we are used to. It’s also part of relaxation.

She continued:

People like to gather, dress nicely, and take photos. They want to do something together. — Above all, they want to meet each other. They did this in Thailand, so they are now also doing it in Finland.

Kamlai also explained that when women engage in Buddhist ceremonies together, they believe they will be reborn together and reunite in the future.

Visiting a temple thus provided an opportunity to connect with individuals of the same cultural background. Kaew, cited above, even described the temple as a “wellness center” where people could find food, company, and free therapy from the monks and each other. She said:

And, if you do not have relatives, if you cannot visit Thailand, if you cannot afford to go home every year, when you go to the temple, you get to speak your mother tongue, you get good food, or you can bring something to sell. There are people who make something delicious or grow vegetables and sell them at the temple. And it’s such a good thing if you cook and share with others. You feel good to take care of others, it’s really natural for us. Even if we do not know other people in advance, when we are there, we have something in common. Language, culture, religion.

Whereas many Thai women who have lived in Finland for a long time come to the temple regularly, the temple seems to hold great significance also for those who have more recently immigrated from Thailand. As most Thais migrate to Finland through marriage, they settle in different parts of the country, often living far away from their compatriots and the Thai community. The temple provides a venue and an opportunity for Thai people to come together, converse in their native languages and dialects, share traditional Thai food, and work together for the temple’s benefit. Although the temple is a religious space, it is also a home-like space where sacred ordinances and everyday chores occur and mix. The temple reminds one of home because it reminds one of Thailand, childhood, and relatives. Upon moving to Finland, a woman may not immediately find her way to the temple. However, during times of hardship, she often seeks solace there.

Communality and relationality at the temple are maintained especially by the act of giving and sharing, which was also highlighted by Kaew above. People visit the temple to offer special tributes on their birthdays or anniversaries of loved ones’ deaths. They bring and cook food for religious occasions and feed monks every single day. Boribun said:

I go to [the temple] to give because Buddhism is much about giving something, and this makes my heart happy. That is why I go to the temple. — The main thing is to give to the monks, and we believe that when we die, we will go to heaven, not to hell. But, of course, in reality, Buddhism is above all about cleaning your heart.

What is interesting is that although giving is emphasized and widely practiced, many women, like Boribun above, noted that giving was only one way to practice Buddhism and earn merit. This apparent contradiction will be discussed in the next chapter.

It was acknowledged that donating to the temple could also lead to financial difficulties. Kaew said:

I’ve also seen people who may not be financially well but want to donate and borrow money from someone. It’s their decision, and maybe they get peace and security for doing this, but I do not think it’s the right way. — Donating is such a big thing, and it’s such a big thing to do something for the temple. You get peace and security, but you can also get into trouble.

People do not just maintain relationships with their living family members and friends at the temple, but they also keep the memory of their deceased loved ones alive by making offerings in their honor. When a family member passes away or on their commemoration day, they take a picture of them and bring it to the temple. Churai said:

We want to go to remember the dead; for example, my father is dead, and I go to the temple and make food that my father liked and send to my father, or Buddha can send to him. Or, when I pray for something, Buddha can send it to my family members who are dead.

It is thus important to note that in addition to sharing physical aspects such as a familiar space, objects, clothing, and food, the women can also share intangible cultural aspects like language, religious beliefs, and customs in the temple. Many women feel homesick and guilty for being far away from their aging parents or children, and relationships with other women and their peer support become crucial. Kaew’s story demonstrates how vital it is to have someone from the same background to share their grief with during times of loss. For all, the monks also hold great significance. Kaew, for example, highlighted the importance of monks and the sense of security they provided to women who were far from home. In the Thai social hierarchy, monks enjoy arguably the highest status, and every Thai man is expected to spend at least a short period of time in a monastery. Monks perform calendar and age-related ceremonies and provide spiritual guidance. When a woman is pregnant, she seeks blessings from a monk for a safe delivery and a healthy child. After birth, the monk gives the child a name. During marriage ceremonies, a monk is present, and when someone moves to a new home or starts a business, they can invite a monk for blessings. Monks also perform the funeral rituals and recite texts for the deceased’s benefit. Moreover, they visit sick or dying people in hospitals or at home. Through monks, it is possible for a layperson to accumulate spiritual merit (p. *puñña*), and that is one of the main reasons why Thais feed and serve monks and finance temples and monastic institutions (Moore, 1974; Kirsch, 1982; Kitiarsa, 2010; Scott, 2017; Swearer, 2003). Unfortunately, Finnish immigration practices have made it difficult for monks to enter and remain in the country, despite their central role and importance.

The temple is not just a physical space, but also a platform for social interactions and relationships. It serves as a hub for people with similar cultural and immigrant backgrounds, where they can come together not only for religious practices, but also to make new friends, share life experiences, and support each other in times of joy and sorrow. The temple provides a sense of home, not only because of its material and symbolic aspects that remind people of their Thai culture, but also because of the people who share the same cultural background, customs, and experiences of immigration. As Kaew mentioned, even though the people who attend the temple are not related, they still feel like relatives and the temple feels like home. Although the need to meet other Thais at the temple may decrease over time, the social aspect of the temple continues to be significant. The human relationships and a sense of community in the temple are maintained in many ways, but food and eating are particularly central (see Plank, 2015). Food is given to monks, but feeding other lay people is also a central way of taking care of others and building trust, acceptance, and communality. The sense of communality and relationality does not extend only to those in the immediate vicinity, but with food, the relationship is also maintained with deceased loved ones.

It can be suggested that the temple, as a network of close social relations, creates a *sense of belonging*, or “the subjective feeling of deep connection with social groups, physical places, and individual and collective experience” (Allen et al., 2021, 87). It is a feeling of being part of a group and developing positive social ties and mutual affective concern (Williams, 2021), or as put by Yuval-Davis (2006, 197), belonging is about emotional attachment, feeling “at home” and “feeling safe”.

### 5.3 Meaning

In Buddhism, the ultimate meaning refers, first, to the inevitability of suffering, which in the Buddhist context means everything from physical and mental pain and distress we feel in the face of impermanence and change to existential angst, and second, to the possibility to overcome suffering.

Although people visit temples for various reasons, the primary purpose is religious or spiritual, and the temple’s significance cannot be understood outside of its religious and soteriological meaning. However, the specific spiritual motivation for each individual may differ. Donating to the monks and the temple, volunteering, and sharing food with both living and deceased individuals are all considered meritorious deeds that can accumulate good karma – or good luck. Additionally, participating in religious celebrations is another way to gain good merit. Chailai explained:

If you want to get good luck, in Thailand, it is customary to go to a temple and give something, money or goods, and not so much cultivate your mind. So many women come [to the temple] to give food and stuff.

She continued:

They do not remember that there are many other ways to get good merit. You do not need a temple. — And then, if someone wants to go a little deeper into Buddhism, do evening chanting and mindfulness training, this is the next step.

Although giving to monks and temples was a popular way to gain merit, women were well aware that there were other ways to accumulate good karma. Dao shared her view this way:

[Women go to the temple] for good luck. First, we believe that offering food to the monks and donating to the temple will bring us good luck. The first thing is thus relationships. So, first, you can give. Second, you can meditate. The third is *samadhi*.^1^ When people do not know much about Buddhism, they think that they should go to the temple and give money and food, thinking that by giving food to the monks, the dead relatives and parents will also get this food, and they themselves will get good luck. This is okay, but it is only the first part.

Dao accepted different reasons and ways to practice Buddhism at the temple, but some women were critical of “cultural” Buddhism’s practice of giving. Ratana was particularly critical among them:

Sometimes people think that they must do many things, help the monks, and go [to the temple] every day. Once, I saw in Thailand that an older lady brought a little bit of food [to the temple] but left with a large amount of food. It’s not just about good food. Religion teaches sharing, and good food and health should be given to everyone. Many Thai people in Finland may also think this way. They donate and then expect to win the lottery. They hope for something for themselves before they give. Religion teaches us to give and only to give. Food is taken to the temple when a parent is dead, and the dead parent gets the food; this is how it is believed. Or it is thought that through meditation, one can achieve some supernatural visions.

She continued:

You can also practice outside the temple. The temple is more about Thai culture. The temple can be a good place for some people; for example, if there are family problems, one can go; the temple is a peaceful place. You can be free from problems for a while. But in fact, you can practice anytime. Many people do not understand this; they think that when you give a lot of money [to the temple], you get something back, but they do not understand that it’s a mental practice.

Dao seemed to agree with this idea when she said:

I do not think it’s even necessary to go to the temple. It can be supported, but the temple is inside your mind. You do not have to go to a temple to get good luck. For example, recycling is enough, because it makes good things happen and makes you feel good. You do not have to do big things; you can start by doing small ones.

She continued:

If you are in pain, you need to go somewhere where you feel you are safe. In the same way, I think, when people go to the temple, they feel they are safe. When people have problems, they believe that by going to the temple, life changes for the better. Even when you believe that you feel better when you go to the temple, you actually feel better. But of course, you do not need to go to the temple to feel good. You can donate to the Red Cross, for example, or to people who are hungry. That makes you immediately happy, it is the same thing.

Churai was also wondering whether Thai people understood the “real” meaning of Buddhism when she said:

At the end, they say, “I give this to win the lottery.” I have a different motivation. I have had mental problems; I go to the temple to thank Buddha for making me feel better. The monk teaches us the right way. I visited a Tibetan temple, and I found that people there had a lot of respect for the Buddha. Maybe my community does not always know the deep meaning of Buddhism. Buddha does not need anything; he only hopes that people have a clear mind.

It was exactly the mind training that most of the women interviewed considered the most essential way of practicing Buddhism. Almost all of them regularly meditated at home. Many were eager to share how their quality of life and perspective had transformed after they began to learn more about Buddhist philosophy and incorporated meditation into their routine. It was also a common practice for many of the women to participate as “nuns” at the temple. These nun retreats are usually organized on weekends, starting on Friday and ending on Sunday. The participants adhere to eight precepts rather than the five guidelines for laypeople. During the retreat, women are dressed in white clothes, and they meditate and listen to Buddhist teachings, but they are also responsible, for example, for cooking for the monks and cleaning the temple. Nevertheless, they thought the retreat provided an opportunity for them to deepen their religious practice, and this practice was believed to be close to the authentic goal of Buddhism, that of cultivating one’s mind in order to overcome ignorance and suffering. Although some women argued that going to the temple for mind training was unnecessary, it was agreed that the temple provided an ideal environment for this purpose. For instance, Ratana mentioned that she did not visit the temple frequently, but she regularly participated in the nuns’ retreats at the temple. She considered the temple to be an appropriate venue for deepening her mind training, and she learned to do it properly there.

Many Thai women living in Finland find solace in visiting the temple, which they view as a refuge from the challenges of daily life. Ratana and Dao both noted that women often seek solace at the temple when they are struggling with personal problems, experiencing pain, or in need of a sense of safety. Churai, on the other hand, visited the temple to express her gratitude for recovering from depression. These sentiments were echoed by Phloi, who also emphasized the temple’s role as a sanctuary for those seeking spiritual comfort:

Religion is part of my life. It affects my mind. If you think about negative things, the monk can tell stories, and you can learn to change your way of thinking. — You learn to let go and not to keep things inside. You can change your mind. You can calm down. You can let go. You can breathe.

Boribun expressed her feelings like this:

Buddhism is peace. I do not disturb anyone. I can feel good and have empathy. If I go to the temple, I feel happy. When I’m happy, others next to me get good energy, too.

These women participated in temple ceremonies and donated their time, money, and food to the temple. Nevertheless, many saw these activities as secondary importance in terms of their spiritual life. Instead, they emphasized the philosophical side of religion as well as cultivating one’s mind through meditation. They also wanted to make a clear difference between “pure Buddhism” and “cultural Buddhism.” However, in everyday life, it is common for Thai people to have different beliefs and ways of practicing religion. They may practice breathing exercises, attend courses on Buddhist philosophy, donate to monks, visit shrines dedicated to spirits, offer prayers in front of Buddha statues, or make offerings to appease the spirits. All of these practices can be observed in combination with each other (Scott, 2017).

Women visited the temple for various religious reasons, resulting in diverse interpretations of Buddhism. However, what was common for all of them was the temple’s ability to provide a safe haven free from problems. Religious activities performed in the temple were believed to bring peace, happiness, and security. Peace of mind and a sense of security were obtained through donating and sharing, which allowed women to accumulate merit and good luck. Additionally, peace of mind and safety were also sought through mind training, which helped individuals understand the impermanence and suffering of life and find ways to overcome it. Both Ratana and Dao mentioned that if people face problems, they can come to the temple. Chailai said she started coming to the temple more often before her divorce. She was unhappy at home, and the temple felt like a “happy place” to be. She had met various women who were going through divorce themselves and got moral support and learned from their stories. Later she also got interested in mind-training. Malee said that she had faced serious workplace bullying, which, in addition to her difficult relationship with her Finnish ex-husband, had led to major depression. However, she had gained a lot of support from Buddhism, especially from the Buddhist mind-training, and felt much better now. Chruai said that she came to the temple because she wanted to thank the Buddha for feeling better after recovering from depression. Thai women living in Finland encounter various challenges concerning their family and social lives. The temple serves for many as a sanctuary where they can confront challenging life situations and emotions and ultimately achieve a sense of temporary relief from them. The central aim of the temple is to provide peace and tranquility that they can carry into their everyday lives. The temple exudes a feeling of safety, and it is not surprising why.

It was suggested above that religion can improve emotional well-being by offering social support and coping resources. It can provide a framework to distance oneself from everyday pressures and allow suppressed emotions to be expressed. Religion can redefine social emotions while supporting societal standards and shaping human character for lasting emotional orientation. Moreover, research has suggested that religious identity and practice can have a positive impact on the mental health of immigrants. Firstly, being part of a religious community can provide them with social and material resources, as well as a sense of belonging, which can help them adjust to dislocation and relocation. Secondly, religion can provide the necessary cognitive and socio-emotional resources to cope with the traumas of migration. Finally, being part of a religious community can also provide immigrants with a network of people from their ethnic background with whom they can continue shared cultural practices and traditions (Wu et al., 2021, 293).

Finally, it has been proposed that religion’s strong influence on individuals’ health and well-being may be due to its ability to provide *meaning*. Religion is a way of understanding and interpreting the world. Like other belief systems, it affects a person’s beliefs, goals, and emotions. However, what sets religion apart is its focus on the sacred and how it shapes an individual’s beliefs, goals, and emotions (Park, 2005, 295; Riis and Woodhead, 2010). The temple embodies the soteriological goal of Buddhism, which is to be free from various forms of suffering.

## 6 Discussion: space, belonging, and meaning

The fact that the temple exists is really important because it reminds us that we also exist. And we are grateful that we have the space to practice our own religion and be ourselves. *Kaew*

The early Thai women’s purpose was to establish a place where Thai individuals could unite and share their life experiences, both happy and sad. One of the founders of the Wat Buddharama temple expressed her happiness and gratitude to me before the temple’s postponed grand opening in June 2022. She shared that the project was not an easy one to start with, considering that in the 1990s, few Thais were living in Finland, and multi-religiousness and multiculturalism were not commonly accepted. It was a challenge for women to find jobs and integrate into Finnish society, and language barriers were also widespread. Despite these obstacles, the temple was finally successfully established.

When viewing the material and immaterial aspects of the temple, it is no wonder why early Thai women in Finland were so eager to establish a temple in Finland and why it remains important to a new generation of Thai people in the country. For these women, the temple serves as a transnational location for material, social, and religious purposes where Buddhism and Thainess are preserved, renewed, and experienced even when far away from their homeland. The temple, as a physical space, serves as a venue for religious activities but also as a material manifestation of Thai culture and religion. It is a communal symbol representing the establishment, growth, and continuation of Thai Buddhism in Finland.

In this article, I have, moreover, argued that the temple provides a sense of safety for many Thai women residing in Finland. The sense of safety of Thai women in their new homeland has not been studied as such. Nonetheless, there is a significant amount of research on the discrimination they face in the Global North, such as workplace bullying and sexual harassment. Studies have also indicated that many Thai women experience physical, mental, and financial violence in their family and marital lives. During this research, women shared their experiences of being bullied, discriminated against, belittled, excluded, and even sexually harassed. These findings suggest that Finnish society and its people may not always provide a sense of safety for Thai women living in the country.

An alternative perspective suggests that Thai temples as transnational religious spaces can provide a sense of safety for many Thai women. However, the significance of the temple for women’s sense of security is often overlooked, particularly in regard to Thai women in a new homeland. As with any emotion, the sense of safety is a complex combination of biological, personal, and private, as well as relational, social, and cultural factors. It develops through our relationship with the environment and people around us. Based on the research on Thai women and temples in Finland, I have suggested here that the temple offers a sense of safety by incorporating and enhancing emotions through its symbolic and material features, communality and relationality, and religious meaning.

As physical places, the temples are communal symbols and sacred domains that are associated with various concepts, symbols, and interpretations. Material things, such as spaces, objects, clothing, food, and sound, serve as mediums that facilitate a variety of uses and experiences and play a crucial role in shaping and defining religious practices, emotions, sensations, and thoughts. The temple is thus not merely a setting for religious activities or a stage for religious experience but an essential part of religious experiences, meanings, and significance.

The temple serves as a foundation for both religious and everyday activities and social interaction between people from the same cultural background and in the same – but also various kinds of – life situations. Its material and non-material aspects, such as its architecture, decorations, clothing, food, sounds, and smells, contribute to this foundation. The culture and traditions that people are familiar with, as well as their interactions and relationships with others who share the same cultural background and experience of immigration, create a sense of belonging. Additionally, the feeling of being seen, heard, and accepted can lead to feelings of security and trust. Women who describe coming to the temple as “going home” or who talk about the peace and happiness they feel at the temple talk about the temple as a place of belonging and where they feel safe.

The temple’s material and non-material aspects, as well as its communality and relationality, work together to connect people with the temple’s religious meaning. Although people may visit the temple primarily for socializing, it is nearly impossible to engage in temple activities without also performing meritorious deeds that are crucial in the Buddhist soteriological path. Offering food or money, praying, chanting, or simply sitting quietly are all considered essential Buddhist practices. All religious practices aim to alleviate the problems and hardships that are faced in daily life. The temple represents this goal and the potential to achieve it. It embodies the concept of ultimate meaning, which in Thai Buddhism acknowledges the inevitability of suffering, but also offers the possibility of overcoming it.

The various emotions and sensations associated with different aspects and functions of the temple can create and promote a sense of safety. The religious space, with its materiality, symbols, relationships, and meanings, intertwines in a complex way, leading to even more complex emotional experiences. One clear sensation that arises from research on Thai women in Finland is a feeling of security or a sense of safety. It is a multifaceted experience as it is partly individual, partly communal, and partly related to the environment.

Nevertheless, it is important to approach the sense of safety that the temple provides with a critical mindset. Although this research suggests – based on the women’s accounts – that Thai women feel safe in the temple, it does not necessarily mean that everyone would have the same experience. While the research participants claim that everyone is welcome and equal in the temple, it is inevitable that some people develop closer relationships with the temple and its monks than others. This is usually influenced by the frequency of visits, having a designated role, or regularly doing voluntary work there. Whether these women are higher up in the temple hierarchy remains unanswered in this study. It is certain that a temple attempting to reconcile different interests cannot be entirely free of conflict. Furthermore, discrimination based on personal qualities such as place of birth, skin color, and wealth is prevalent in Thai culture, and it is possible that it also occurs in the temple. It is also worth considering whether the temple isolates women from Finnish society by making it easier for them to connect with a community that speaks the same language and shares the same culture than to reach out to the wider Finnish society. However, it is important to note that the temple does not hinder women from participating in Finnish society nor prevent them from, for example, converting to Christianity. While this study cannot answer the question of isolation, it is safe to assume that separation depends on various factors other than the temple, including how well women are, in general, integrated into Finnish society. This integration usually depends on how long they have lived in Finland.

At the beginning of this chapter, Kaew highlights that the temple signifies the presence of often invisible Thai Buddhist women in Finland. For the research participants, the temple serves as a space for religious and social activities, evoking personal and shared meanings, emotions, sensations, and belonging through its physical and social elements. The temple embodies this shared experience and provides the ultimate meaning and a sense of safety. These considerations speak for the fact that when facing the problems of Thai women or other religious and ethical minorities, it is necessary to also look to their religion as a source of mental and spiritual well-being.

## Data availability statement

Requests to access the datasets should be directed to mitra.harkonen@helsinki.fi.

## Ethics statement

Ethical review and approval was not required for the study on human participants in accordance with the local legislation and institutional requirements. The studies were conducted in accordance with the local legislation and institutional requirements. The participants provided their written informed consent to participate in this study. Written informed consent was obtained from the individual(s) for the publication of any potentially identifiable images or data included in this article.

## Author contributions

MH: Writing – original draft, Writing – review & editing.

## Footnotes

^1^A state of meditative consciousness.
